# Supplementary figures and images for: Comparisons of the Oral Microbiota from Seven Species of Wild Venomous Snakes in Taiwan Using the High-Throughput Amplicon Sequencing of the Full-Length 16S rRNA Gene
Source: Biology (Basel). 2023 Sep 4;12(9):1206. doi: 10.3390/biology12091206 (PMC10525742; doi:10.3390/biology12091206)

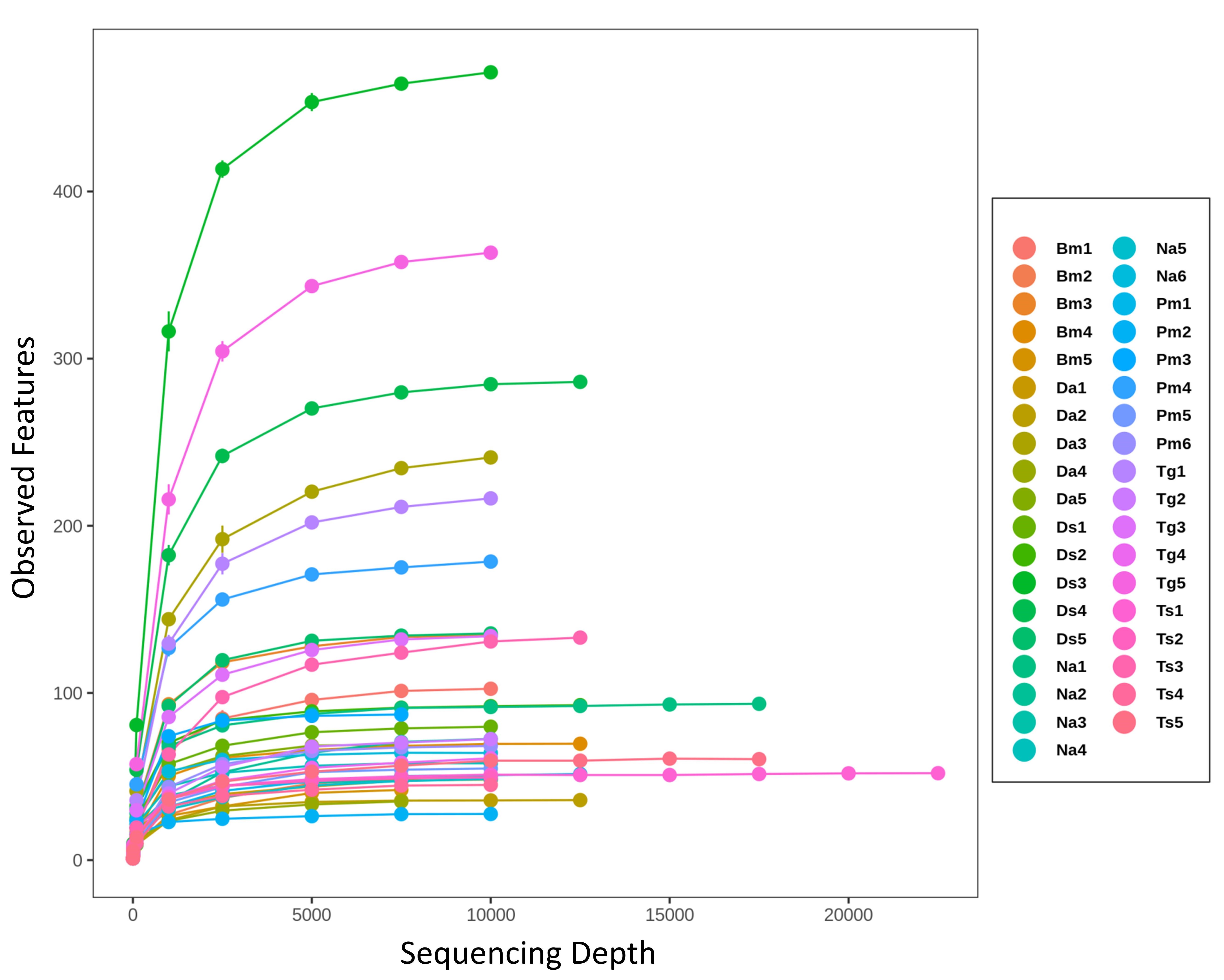

Supplement: Supplementary file 1 [file biology-12-01206-s001.zip › Supplementary Figure S1.jpg]
